# Bleeding Isolated Gastric Varices as a Rare Presentation of Pancreatic Neuroendocrine Tumor: Case Report and Literature Review

**DOI:** 10.7759/cureus.9670

**Published:** 2020-08-11

**Authors:** Sreeja Sompalli, Saif Faiek, Margaret Mallari, Jacinto Camarena

**Affiliations:** 1 Internal Medicine, AtlantiCare Regional Medical Center, Atlantic City, USA; 2 Vascular Institute/Interventional Radiology, Atlantic Medical Imaging, Galloway, USA; 3 Radiology/Interventional Radiology, AtlantiCare Regional Medical Center, Atlantic City, USA

**Keywords:** gastric varices, isolated gastric varices, pancreatic neuroendocrine tumors, bleeding gastric varices

## Abstract

Isolated gastric varices (IGV) are gastric varices in the absence of esophageal varices. IGV is one of the rare causes of gastrointestinal bleeding and an uncommon complication of pancreatic neuroendocrine tumors (PNET). The gold standard diagnostic tool of varices is esophagogastroduodenoscopy (EGD). IGV tend to bleed with lesser portal pressure compared to esophageal varices. Initial treatment is similar to the gastroesophageal varices. The intervention options include endoscopic, radiological, and surgical approach.

## Introduction

Isolated gastric varices (IGV) can develop with or without the formation of esophageal varices. IGV occurs less frequently than esophageal varices; however, it has a higher risk of rebleeding, a rapid progression, and higher mortality [1]. Portal hypertension due to liver cirrhosis is the most common cause of both gastroesophageal varices and IGV. In noncirrhotic patients, IGV occurs secondary to localized portal hypertension due to splenic vein obstruction [2]. According to Sarin's classification, IGV can be divided into two types, depending on the location [3]. In type I (IGV1), varices are located in the gastric fundus. Type II (IGV2), including gastric varices, may develop in any other area of the stomach [4]. Knowing the location of varicosities, gastroesophageal versus IGV, has significant implications for treatment strategies [1].

## Case presentation

**Figure 1:**
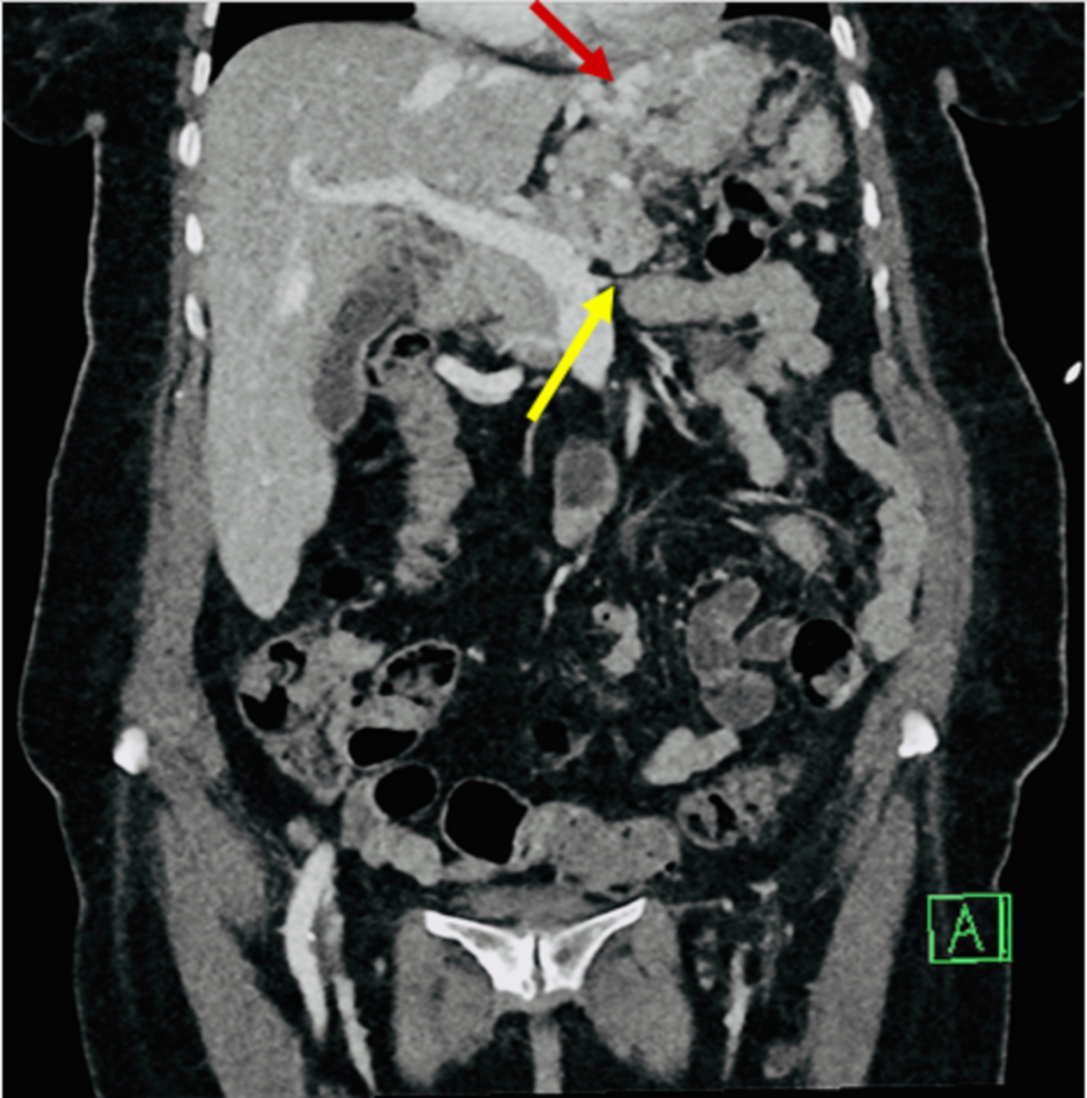
Coronal abdominal computed tomography (CT) scan Portovenous delay showing gastric fundal varies (red arrow) and termination of splenic vein circulation (yellow arrow) secondary to thrombosis

**Figure 2:**
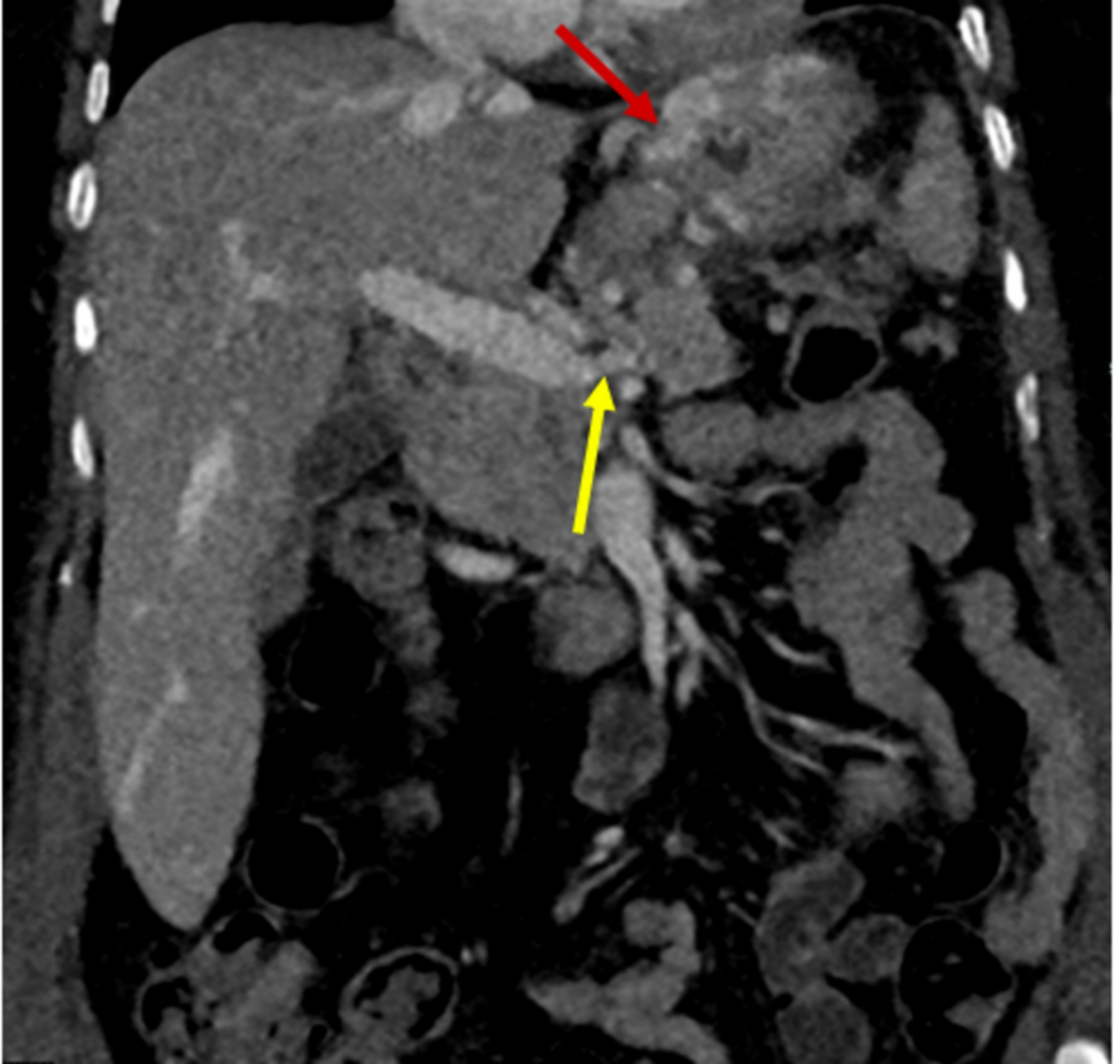
Coronal abdominal computed tomography (CT) scan Portovenous delay showing gastric fundal varices (red arrow) and termination of splenic vein circulation (yellow arrow) secondary to thrombosis

**Figure 3:**
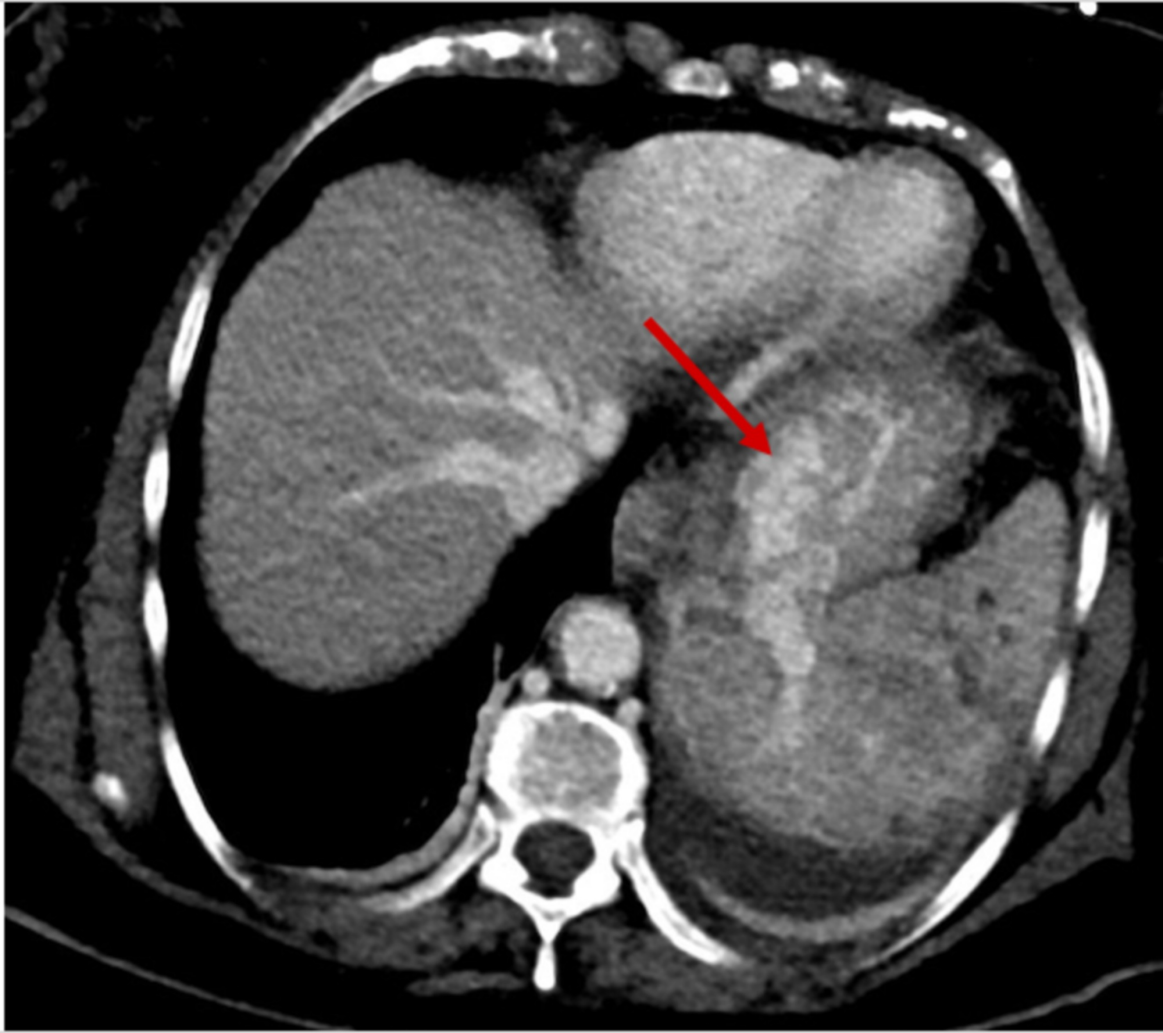
Axial abdominal computed tomography (CT) scan Portovenous delay showing gastric fundal varices (red arrow)

A 71-year-old Hispanic woman with a past medical history of hypertension, hyperlipidemia, and diabetes mellitus presented to the hospital with an episode of hematemesis, generalized weakness, and epigastric abdominal pain for two days duration. Laboratory studies on admission were remarkable for normocytic anemia and a positive fecal occult blood test. The patient was admitted to the hospital and started on intravenous (IV) Protonix® (pantoprazole sodium) (Pfizer, Inc., New York, NY, USA). Her hemoglobin continued to plummet, and as a result, she required multiple blood transfusions. The gastrointestinal (GI) service evaluated the patient. An upper endoscopy was performed which revealed a bleeding ulcer in the gastric fundus that was subsequently treated successfully with Hemospray® (Cook Medical, Winston-Salem, North Carolina, USA) therapy. Computed tomography (CT) of the abdomen/pelvis with contrast demonstrated an infiltrating mass involving the pancreatic tail and the spleen, causing splenic vein thrombosis and large gastric fundal varices (Figures 1-3). 

A left hepatic lobe mass was also identified on imaging and the pathologic specimen from a percutaneous biopsy. The pathology sections showed focal benign hepatic parenchyma and nests of tumor cells with papillary architecture and nuclear pleomorphism. No mitotic figures were present in 10 high-power field count; tumor cells were positive for pankeratin, chromogranin, synaptophysin, and cluster of differentiation (CD)56. Alpha-1-antichymotrypsin (A-ACT) and CD10 were equivocal with high background staining, negative for arginase 1 and leukocyte common antigen (LCA), beta creatinine did not show nuclear staining, and Ki-67 was ~3%. These findings favored a well-differentiated neuroendocrine tumor. 

After an extensive multidisciplinary meeting, the patient did not opt for aggressive treatment. In the setting of substantial tumor burden, the patient underwent a short course of radiation therapy and was started on octreotide infusion to prevent any further upper GI bleeding. The patient was then discharged and a follow-up outpatient visit with the oncology service was arranged.

## Discussion

Pancreatic neuroendocrine tumors (PNET) are sporadic and diagnostically challenging tumors with a wide variety of clinical presentations [5]. IGV is one of the uncommon causes of gastrointestinal bleeding and a rare complication of PNET. PNET may compress or invade adjacent vasculature and cause an obstruction. Due to the non-specific manifestations, the diagnosis of PNET may be delayed until metastasis occurs. 

Our patient presented with acute upper gastrointestinal bleeding secondary to metastatic PNET. An abdominal CT scan demonstrated an infiltrative mass involving the pancreatic tail and spleen, causing splenic vein thrombosis and regional portal hypertension, leading to type 1 IGV. In this case, IGV1 resulted from the redirection of blood flow through the short gastric veins [6]. IGV1 constitutes less than 2% of all gastric varices. The classification of gastric varices has significant implications regarding the risks of bleeding and management. The incidence of bleeding is higher in IGV1 (78%) compared to IGV2 (10%) [7].

The most common cause of IGV is portal hypertension secondary to liver cirrhosis after the obliteration of esophageal varices. Bleeding IGVs usually occur secondary to the isolated splenic vein obstruction due to pancreatic, gastric, colon, and, rarely, renal carcinoma.

Esophagogastroduodenoscopy (EGD) is the gold standard for diagnosing gastroesophageal varices [8]. In our patient, IGV was diagnosed via EGD. The splenic vein thrombosis secondary to the infiltrative tumor was discovered on the CT of the abdomen/pelvis. There is limited literature on specific treatment strategies for IGV1. The initial management for actively bleeding IGV1 is similar to that of esophageal varices, including early administration of vasoactive drugs, prophylactic antibiotics, early replacement of volume, and maintaining adequate volume status. The threshold for bleeding tends to occur at a lower portal pressure for IGV compared to esophageal varices. Hence, more potent vasoactive drugs and endoscopy are recommended to control bleeding from IGV1 [7]. The options for intervention include endoscopic, radiological, and surgical approaches. A review of the literature shows that endoscopic therapy with tissue adhesives, mainly cyanoacrylate (CA), is widely used for acute bleeding from IGV1 [9]. Repeated sessions with CA injections, along with nonselective beta-blocker use, are recommended after initial hemostasis is achieved [10]. Transjugular intrahepatic portosystemic shunt (TIPS) creation was shown to be a successful salvage therapy in uncontrolled bleeding in patients with liver cirrhosis or portal vein thrombosis [11]. However, in our patient, TIPS was not done since there was no evidence of liver cirrhosis. 

Other options include transarterial embolization of the splenic artery, balloon-occluded retrograde transvenous obliteration (BRTO), or any of its variations, and splenic resection. The other variations of BRTO include coil-assisted retrograde transvenous obliteration (CARTO) and plug-assisted retrograde transvenous obliteration (PARTO) [1, 12]. The BRTO and its variations are the treatment of gastric varices only in the presence of a gastro renal shunt. Splenectomy or splenic artery embolization are good options for patients with left-sided portal hypertension due to splenic vein thrombosis with recurrent IGV bleeding [7, 13]. Our patient did not opt for extensive surgical resection of the tumor. Therefore, we offered a short course of radiation therapy and octreotide infusion to prevent recurrent gastric mucosal bleeding.

## Conclusions

PNET presenting with bleeding IGV is uncommon. In patients presenting with IGV without risk factors or history of liver cirrhosis, it is crucial to consider other causes of regional portal hypertension, such as splenic vein thrombosis and obstruction, and the possibility of underlying malignancy.
